# Structure diversity of nitric oxide synthases (NOS): the emergence of new forms in photosynthetic organisms

**DOI:** 10.3389/fpls.2013.00232

**Published:** 2013-07-04

**Authors:** Natalia Correa-Aragunde, Noelia Foresi, Lorenzo Lamattina

**Affiliations:** Instituto de Investigaciones Biológicas - CONICET, Universidad Nacional de Mar del PlataMar del Plata, Argentina

## NO synthesis from mammalian and photosynthetic organisms

Humans have enormously increased the level of nitrogen (N) circulating in the troposphere and the earth surface during the last century, correlating with the population increase. As an undesirable consequence, high levels of reactive N are polluting the environment where humans inhabit. Nitric oxide (NO) is one of the reactive N species with both positive and negative impact on life. NO synthases (NOSs) are enzymes that oxidize arginine to citrulline and generate the denitrifying intermediate NO which can be subsequently reduced to N_2_O and N_2_. NOS are large modular enzymes present in all kingdoms which through evolution were the result of multiple gene and genome duplication events together with changes in protein architecture (Andreakis et al., 2011). A recently described NOS from the marine unicellular microalgae *Ostreococcus tauri*, belonging to the picoplankton in oceans, adds new insights to study the evolution of the complex organization of these enzymes. In this opinion we discuss the structure diversity of the emerging new NOS forms described in prokaryotes and eukaryotes. Regarding the controversy about the existence of canonical NOS in higher plants, we propose that the latest findings support the existence of a high diversity of NOS forms in different lineages. Thereby, since higher plant species whose genomes have been fully sequenced, which are scarce, it cannot be discarded that a new form of NOS may have evolved in higher plants.

**Figure 1:**
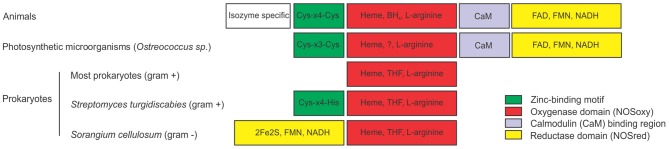
**Structures of nitric oxide synthases (NOSs) from different sources**. Comparison of animal NOS structure with NOSs from photosynthetic microorganisms and prokaryotes. Animal NOSs contain a zinc-binding region (Cys-X4-Cys), a NOS oxygenase domain (NOSoxy) which binds Heme, arginine and BH_4_, a calmodulin-binding region (CaM) and a NOS reductase domain (NOSred), which binds FMN, FAD, and NAD. The only described NOS of the photosynthetic organism is from the Ostreococcus genus. It has the NOSoxy, NOSred, a partially conserved CaM domain and a Zn-binding motif that partially differs from animals (Cys-X3-Cys). Most prokaryotes has only the NOSoxy domain, with the exception for the gram negative bacterium *Sorangium cellullosum* that has a novel NOSred domain in the N-terminal of the protein containing a 2Fe2S ferredoxin subdomain. *Streptomyces turgidiscabies* also has a partially conserved zinc binding motif (Cys-X4-His). Most prokaryotes produce tetrahydrofolate (THF) instead of the cofactor BH_4_. ? indicates that the co-factor that replaces BH_4_ in Ostreococcus is unknown.

Mammalian NO synthases (NOSs) were the first NOS structures to be biochemically characterized, crystallized and their complete structure deciphered by X-ray diffraction. These NOSs are functional as homodimers, each monomer consisting on an N-terminal oxygenase domain (NOSoxy) containing the binding sites for the cofactors heme, tetrahydrobiopterin (BH4) and for the substrate L-arginine, and a C-terminal reductase domain (NOSred) that binds to NADPH, FAD and FMN (Figure 1). Mammalian NOSs require the binding of calmodulin (CaM) for electron transfer from reductase to oxygenase domain. NOSred has strong sequence similarity with NADPH cytochrome P450 reductase (Stuehr, 1999; Alderton et al., 2001). The crystal structure of NOS showed that there is a zinc tetrathiolate center in the dimeric form of NOS. The zinc ion is coordinated by 4 Cys, two from each subunit. The Cys-X_4_-Cys motif involved in Zn coordination seems to be conserved in all animal NOS described so far (Figure 1). The NOSoxy and NOSred domains have been separately cloned and expressed as recombinant proteins without alteration of their catalytic properties (McMillan and Masters, 1995; Chen et al., 1996). Particularly in mammals, there are three distinct NOS isoforms: neuronal (nNOS), endothelial (eNOS), and inducible (iNOS) encoded by three different genes. These three isoforms differ in localization, regulation and catalytic properties (Alderton et al., 2001). The isolation and characterization of NOS proteins from different species from different kingdoms indicate that NOSs differ structurally and biochemically. Moreover, it has been suggested that NOS could catalyze different reactions depending on substrate and cofactors concentrations (Weaver et al., 2005).

The recent identification of the NOS from the green algae *Ostreococcus tauri* represents the first NOS characterized in photosynthetic organisms (plant kingdom) (Foresi et al., 2010). Ostreococcus NOS (OtNOS) has a 42% of similarity to human NOS reaching to 45–49% similarity to invertebrate NOS. OtNOS contains the NOSoxy and NOSred domains joined by a CaM binding domain (Figure 1). Despite the high similarity, some differences could be noted in the structure of the OtNOS with respect to animal NOS. CaM plays a critical role in activating NOS, since it triggers the electron transfer from flavin to heme. In eNOS and nNOS the electron transfer is triggered by CaM binding while in iNOS, CaM is irreversibly bound. That explains why iNOS is active independently of Ca^2+^ concentration. Indeed OtNOS activity behaves like an intermediate between eNOS/nNOS and iNOS isoforms since in the absence of Ca^2+^-CaM, OtNOS retains almost 70% of activity. Furthermore, OtNOS lacks of the autoregulatory control element (ACE) (Foresi et al., 2010), indicating that it is close to the mammalian iNOS isoform. The ACE impedes CaM binding and enzymatic activation in constitutive NOSs. The increase in Ca^2+^ concentration triggers the binding of Ca^2+^-CaM in constitutive NOS by displacing the ACE (Salerno et al., 1997). The Zn binding motif Cys-X_3_-Cys in OtNOS is partially conserved compared to Cys-X_4_-Cys in mammalian NOS. Even though the binding of Zn to OtNOS has not been experimentally proved, there are other examples of Zn binding motif consisting of Cys-X_3_-Cys (Barbosa et al., 1989; Vasak and Hasler, 2000). BH_4_ cofactor is essential for NO production in animals since the absence of BH_4_ uncouples the reaction leading to NADPH oxidation and superoxide formation. Ostreococcus genome has been completely sequenced (Derelle et al., 2006) and it lacks the genes encoding for the enzymes that synthesize BH4, suggesting that OtNOS may bind another cofactor for catalytic activity.

## Diversity of NOS structure in prokaryotes

Most bacterial NOSs have been described in Gram-positive bacteria and consist of the NOSoxy domain lacking of the C-terminal NOSred domain (Figure 1). NOSoxy from bacteria are similar to animal NOSoxy (Crane et al., 2010). Several studies indicate that bacterial NOS use redundant cellular reductases as electron donors for the catalytic activity (Gusarov et al., 2008). As in Ostreococcus, most bacteria do not synthesize the cofactor BH_4_ and thereby, they probably use tetrahydrofolate (THF) required for NOS activity (Adak et al., 2002a,b). NOSs from bacteria do not contain the CaM binding motif (Crane et al., 2010). Actually, CaM has not been identified in bacteria suggesting that CaM domain is indeed exclusive for eukaryotic NOS. Most bacterial NOS lacks tetrahedral zinc center, with the exception of NOS from *Streptomyces turgidiscabies*, where one of the two Cys is conserved and the other is replaced by His (Kers et al., 2004). Bacterial NOSs also work as homodimers. Excitingly, the discovery of NOS from the Gram-negative bacteria *Sorangium cellulosum* (scNOS) resulted in a different and novel NOS structure. ScNOS is the only characterized bacterial NOS with a covalently attached reductase domain (NOSred). This reductase module has a 2Fe2S ferredoxin domain, a FAD- binding motif and a NAD-binding motif. Interestingly, scNOS has an inverted structure: the NOSred domain is located at the N-terminal and NOSoxy at the C-terminal (Agapie et al., 2009). A similar NOS structure was found in the cyanobacteria *Microcoleus vaginatus* and *Crinalium epipsammum* (accession number ZP_08493682 and YP_007142230 respectively), although it still remains to be confirmed the NOS activity of these proteins.

The lack of apparent NOS in the plants which are most commonly worked on has led to suggest that either plants have lost this gene in the course of evolution or the gene has strongly diverged to a yet unknown new type of NOS. Since several evidences support an arginine-dependent NO production in higher plants reminiscent of a NOS activity (Cueto et al., 1996; Caro and Puntarulo, 1999; Simontacchi et al., 2004; Corpas et al., 2006, 2009; Flores et al., 2008), more efforts should be made to identify this elusive NOS form. It is noteworthy that among the NOS structures described so far, few differences were detected in the NOSoxy domain indicating that might be the core of the enzyme. Therefore the search of new NOS isoforms that differ in the NOSoxy domain would probably be the key to unravel the molecular evolution of this domain and the presence of this protein in the plant kingdom.

Overall, the unexpected diversity of the NOS structures that are currently reported in the literature should allow us to keep optimistic for identifying the NOS gene/s or protein/s complex responsible of NO generation from L-arginine in higher plants.
